# Degenerative Grade 3 Spondylolisthesis Management: A Case Report and Literature Review

**DOI:** 10.7759/cureus.29374

**Published:** 2022-09-20

**Authors:** Gerald Musa, Rossi E Barrientos Castillo, Mihail V Slabov, Kondwani Chirwa, Gennady E Chmutin, Manuel de Jesus Encarnacion Ramirez, Renat Nurmukhametov

**Affiliations:** 1 Neurosurgery, People's Friendship University of Russia, Moscow, RUS; 2 Neurological Surgery, People's Friendship University of Russia, Moscow, RUS; 3 Neurological Surgery, City Clinical Hospital named after C.C. Yudina, Moscow, RUS; 4 Orthopaedics and Trauma, University Teaching Hospital, Lusaka, ZMB; 5 Neurological Surgery, People's Friendship University of Russia (RUDN University), Moscow, RUS

**Keywords:** lumbar spine, meyerding classification, plif, spondylolisthesis, degenerative spine diseases

## Abstract

Degenerative spine disorders are very common in the aging population. Degenerative spondylolisthesis is a relatively uncommon cause of chronic back pain in these patients. We present a case of high-grade spondylolisthesis managed with posterior lumbar interbody fusion (PLIF) and reduction of the listhesis with excellent results. A 56-year-old woman presented with chronic lower back pain managed as an outpatient for over 5 months with no relief. She had no history of trauma or risk factors for malignancy. Lumbosacral CT showed Meyerding grade 3 anterolisthesis of the fifth lumbar vertebra with complete L5-S1 disc collapse and bilateral spondylolysis. An MRI confirmed the findings. There was no spinal canal stenosis. The patient was managed with L4-L5-S1 transpedicular fixation and L5-S1 interbody cage with reduction of the listhesis. The patient had an incidental intraoperative dural tear which was repaired primarily and a wound drain was kept for 5 days without complications. The patient was ambulating by day 5 and was discharged without complications on day 10. Degenerative spondylolisthesis can cause chronic back pain with or without a history of trauma. Although no specific clinical features exist for this condition, it should be suspected in elderly patients even in the absence of a history of trauma. Surgical management in high-grade spondylolisthesis is indicated with interbody fixation and reduction.

## Introduction

Degenerative spine disorders are very common in the aging population. Degenerative spondylolisthesis is a relatively uncommon cause of chronic back pain in these patients. However, the most common presentation in spondylolisthesis is back pain. Spondylolisthesis is the translation of the vertebral body over the adjacent lower vertebra. Depending on the direction of the translation, it is classified as anterolisthesis (anterior displacement) or retrolisthesis (posterior displacement) which occurs with or without pars interarticularis fracture [1-4]. According to the literature, the segment most affected by degenerative anterolisthesis is L4-L5, and it occurs more frequently in women than in men [3]. However, high-grade listhesis is more common at the L5-S1 level [3]. The physiological curves of the vertebral column are very important as they absorb the forces of both physiological and pathological stress and also serve as a lever, thus protecting the spinal cord and nerve tissue [2-3]. When normal spinal alignment is affected as seen in spondylolisthesis, it can lead to compression, traction, or shear injury [2]. The literature indicates that sagittal spinopelvic equilibrium plays a vital role in the development and progression of spondylolisthesis. The stability of the lumbar segment is determined by both clinical and radiological criteria [5]. High-grade spondylolisthesis defined as translation >50% is an uncommon cause of low back pain in adults which may sometimes present with radiculopathy. High-grade spondylolisthesis is seen in 11.3% of patients with listhesis [6]. The clinical presentation will depend on the degree of displacement of the vertebra [5, 7-8]. 

## Case presentation

**Figure 1:**
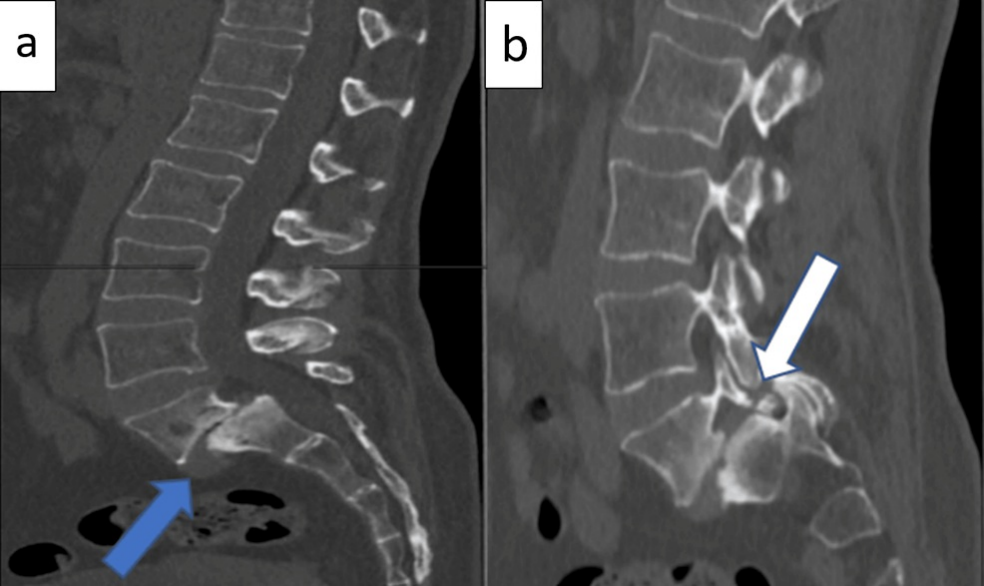
Preoperative sagittal cut CT scan images. a: shows grade 3 spondylolisthesis of L5 (blue arrow) and complete loss of the L5-S1 disc space with reactive sclerosis of the endplates. b: shows spondylolysis of L5 (white arrow).

**Figure 2:**
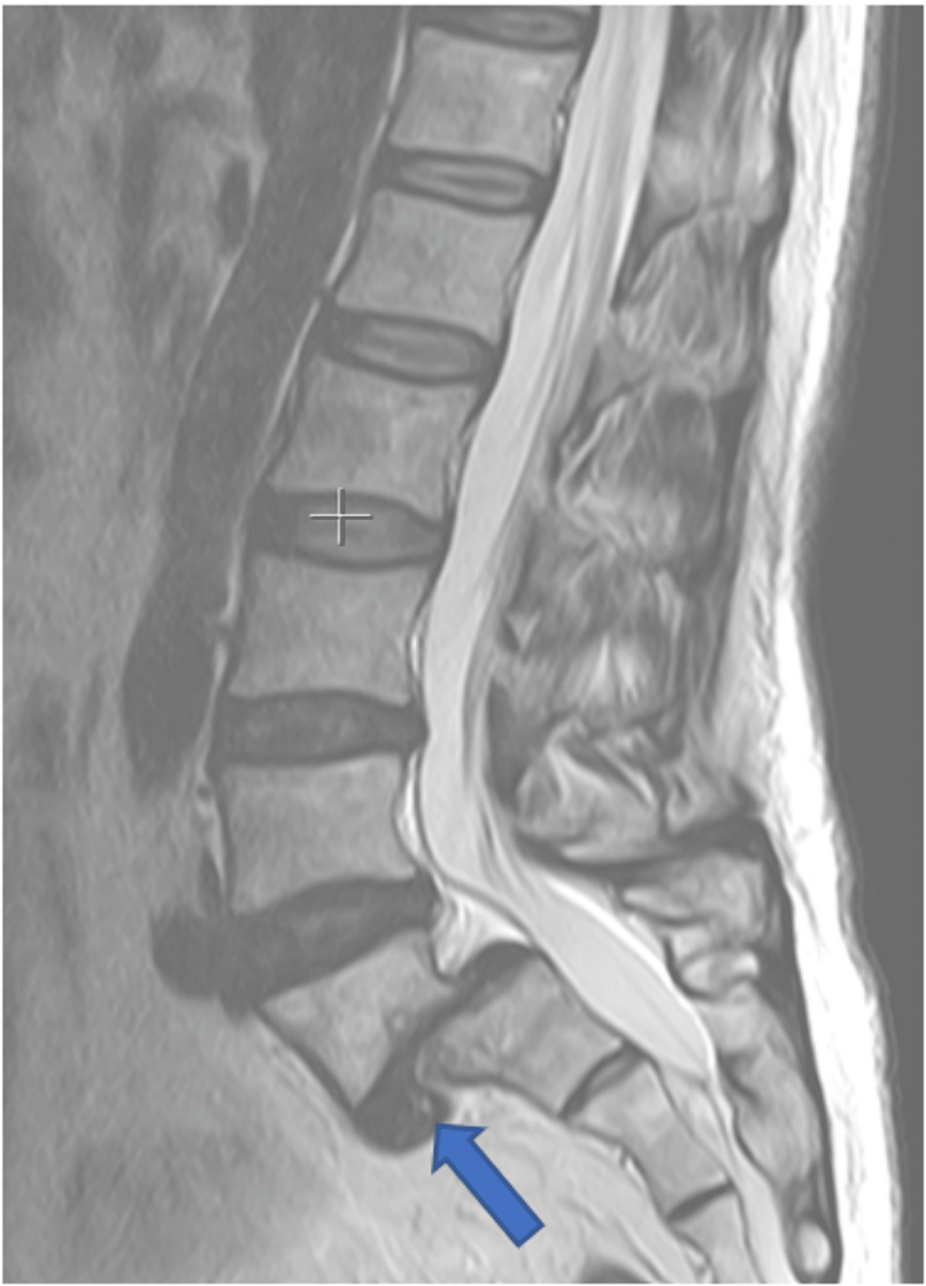
Preoperative sagittal MRI. It shows the L5 anterolisthesis with complete disc collapse and anterior disc herniation (blue arrow) with no modic changes. There was no stenosis of the spinal canal.

A 56-year-old woman presented to the hospital with a persistent backache. There was no history of recent trauma. Her medical history was nonrevealing with no identifiable risk factors for pathological osteoporosis or malignancy. She was managed as an outpatient for the pain for 5 months with no considerable improvement. On neurological examination, she had muscle tension and spasms of the lower lumbar muscles with no radicular symptoms. A CT scan performed showed an anterolisthesis of L5 on S1 with disc collapse and bilateral pars fractures (Figure 1). An MRI was performed to assess the neural structures (Figure 2).

 

The patient was admitted and scheduled for a planned posterior transpedicular fixation. Following preoperative workup and relevant consultation, the patient underwent surgery on day 3.

Operative technique

The patient was in a prone position with adequate padding of the pressure points. The hips and knee joints were flexed to facilitate exposure L5. A midline incision was made from L3 to S2. The approach to the vertebral column and screw insertion into L4-L5-S1 was performed in a standard fashion. An L5-S1 laminectomy was performed. The S1 nerve root foramina were maximally decompressed and the nerve roots were freed to allow for maximal dural sack manipulation without extensive traction on the nerve roots. L5-S1 discectomy was performed with maximal disc removal laterally. Reduction of the listhesis was attempted and done satisfactorily. During reduction, there was a small iatrogenic dural tear which was repaired primarily. The rest of the closure was according to the classic standard, in layers. A drain was left in place for 5 days.

**Figure 3:**
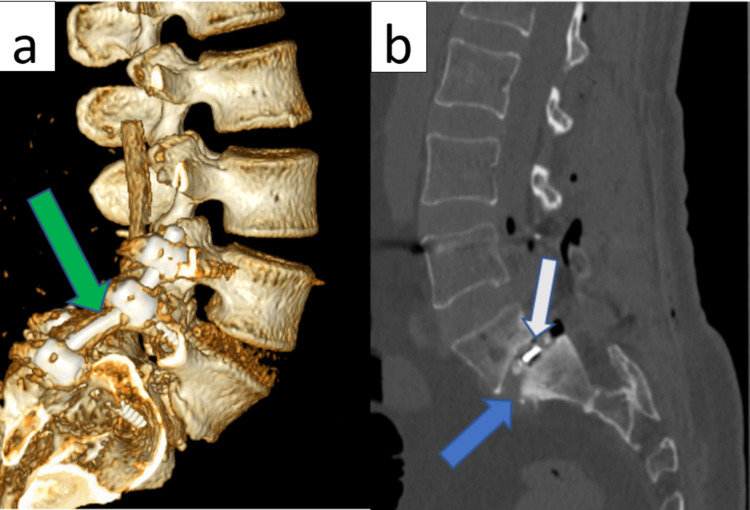
Postoperative CT control images. a: showing a CT 3D reconstruction with the six screws transpedicular fixation in L4-L5-S1 (green arrow). b: shows the L5-S1 interbody cage (white arrow) with adequate reduction of the listhesis and restoration of the height of the L5-S1 disc (blue arrow).

There were no postoperative complications. The patient was ambulating by day 5 and was discharged on day 10 postoperatively. The control CT scan revealed expected postoperative changes as well as acceptable reduction. Subsequent reviews at 1 month and 2 months postoperatively were unremarkable. The patient had no complaints and the back pain had resolved by the second month (Figure 3).

## Discussion

Radiological studies, such as an upright standing spine X-ray, can be used to assess changes in the anatomy of the spinal column that are not visible in the supine position [9]. However, Zhou et al. concluded that the combination of natural sitting and supine sagittal MRI was suitable for the traditional flexion-extension modality for assessing translational instability in patients with degenerative lumbar spondylolisthesis [10]. MRI remains the gold standard for assessing neural structural compression [1].

In many cases, spondylolisthesis is due to an anatomical defect of the pars interarticularis [8]. In this case, the patient showed bilateral pars interarticularis fractures with obvious stress changes on adjacent levels indicating instability and age-related degeneration. In view of these findings, the patient was diagnosed with degenerative spine disease and isthmic spondylolisthesis. The Meyerding classification classifies it as high grade (more than 50% displacement) and low grade (less than 50%) and further subclassifies it into five grades depending on the percentage of displacement. Grade I corresponds to 25%, grade II from 26% to 50%, grade III from 51% to 75%, grade IV ranges from 76% to 100%, and grade V has more than 100% displacement also known as spondyloptosis [4, 8]. Another classification that depends on the etiology, the Wiltse-Newman classification classifies spondylolisthesis into six types: type I or dysplastic, type II or isthmic, type III or degenerative, type IV or traumatic, type V pathological, and type VI or iatrogenic. Wiltse concludes that the isthmic and degenerative spondylolisthesis are the most severe types [1, 4, 8]. Another system, Marchetti and Bartolozzi, merges the dysplastic and isthmic types of the Wiltse-Newman system and broadly classifies spondylolisthesis as either developmental or acquired [8]. Both systems describe the etiological aspects of spondylolisthesis, hence they are not very useful for surgical treatment planning. 

For surgical planning, the Spinal Deformity Study Group (SDSG) classification is usually used, which proposes six sagittal postures based on the radiographic measurement of the degree of displacement and spinopelvic alignment. This system was built for the surgical planning of the spondylolisthesis of the L5-S1 level and classifies spondylolisthesis into a low grade (less than 50%) and a high grade (more than 50%) [8]. 

Anterolistesis is associated with neurological deficits such as foot drop and other motor disorders of L5 [2, 4-5]. In this case, the patient had no radiculopathy but presented with chronic backache. An interbody fusion is not always performed, in some cases, only transpedicular fixation is performed giving excellent results without neurological deficits. The reduction of anterolisthesis today can be achieved with modern techniques, currently, it is done by incorporating interbody fusion techniques. The goal is to achieve stable arthrodesis and reduce kyphosis. One of the risks of reducing the listhesis is an iatrogenic neurological deficit which may persist in the postoperative period [8]. Literature suggests that the concepts of sacropelvic and spinopelvic balance are invaluable in developing surgical techniques [3, 8]. In this case, the decision to reduce the listhesis was based on the high Meyerding grade and sagittal imbalance, and hypermobility of this segment. The reduction was achieved through the distraction of L4 and S1 and maximal L5-S1 discectomy and nerve root canal enlargement, to reduce the deformity, and have a clearer visualization of the L5 screw entry point. 

Although some authors consider conservative or chiropractic management to be viable options [2-3], the majority of studies show that decompression plus fusion is the best course of treatment with better clinical and radiological outcomes, though the surgical techniques that can be used to treat high-grade lumbar spondylolisthesis are still being debated [3-4,10-12]. Some authors have demonstrated favorable results with the modified Bohlman and reverse Bohlman technique for the treatment of high-grade spondylolisthesis [4].

## Conclusions

Degenerative spondylolisthesis can cause chronic back pain with or without a history of trauma. Although no specific clinical features exist for this condition, it should be considered in elderly patients even in the absence of a history of trauma. Flexion-extension images and MRI are invaluable in the diagnosis of this condition. Surgical management of high-grade spondylolisthesis is usually done with interbody fixation and reduction. 
